# Identification of *Candida glabrata* Genes Involved in pH Modulation and Modification of the Phagosomal Environment in Macrophages

**DOI:** 10.1371/journal.pone.0096015

**Published:** 2014-05-01

**Authors:** Lydia Kasper, Katja Seider, Franziska Gerwien, Stefanie Allert, Sascha Brunke, Tobias Schwarzmüller, Lauren Ames, Cristina Zubiria-Barrera, Michael K. Mansour, Ulrike Becken, Dagmar Barz, Jatin M. Vyas, Norbert Reiling, Albert Haas, Ken Haynes, Karl Kuchler, Bernhard Hube

**Affiliations:** 1 Department of Microbial Pathogenicity Mechanisms, Leibniz Institute for Natural Product Research and Infection Biology – Hans Knoell Institute, Jena, Germany; 2 Integrated Research and Treatment Center, Sepsis und Sepsisfolgen, Center for Sepsis Control and Care (CSCC), University Hospital, Jena, Germany; 3 Department of Medical Biochemistry, Max F. Perutz Laboratories, Medical University, Vienna, Austria; 4 College of Life and Environmental Sciences, University of Exeter, Exeter, United Kingdom; 5 Massachusetts General Hospital, Department of Medicine, Division of Infectious Disease, Boston, Massachusetts, United States of America; 6 Laboratoires Constant Burg, Paris, France; 7 Institute for Transfusion Medicine, University Hospital, Jena, Germany; 8 Division of Microbial Interface Biology, Research Center Borstel, Leibniz Center for Medicine and Biosciences, Borstel, Germany; 9 Institute for Cell Biology, University of Bonn, Bonn, Germany; 10 Friedrich Schiller University, Jena, Germany; Institute of Microbiology, Switzerland

## Abstract

*Candida glabrata* currently ranks as the second most frequent cause of invasive candidiasis. Our previous work has shown that *C. glabrata* is adapted to intracellular survival in macrophages and replicates within non-acidified late endosomal-stage phagosomes. In contrast, heat killed yeasts are found in acidified matured phagosomes. In the present study, we aimed at elucidating the processes leading to inhibition of phagosome acidification and maturation. We show that phagosomes containing viable *C. glabrata* cells do not fuse with pre-labeled lysosomes and possess low phagosomal hydrolase activity. Inhibition of acidification occurs independent of macrophage type (human/murine), differentiation (M1-/M2-type) or activation status (vitamin D_3_ stimulation). We observed no differential activation of macrophage MAPK or NFκB signaling cascades downstream of pattern recognition receptors after internalization of viable compared to heat killed yeasts, but Syk activation decayed faster in macrophages containing viable yeasts. Thus, delivery of viable yeasts to non-matured phagosomes is likely not triggered by initial recognition events via MAPK or NFκB signaling, but Syk activation may be involved. Although V-ATPase is abundant in *C. glabrata* phagosomes, the influence of this proton pump on intracellular survival is low since blocking V-ATPase activity with bafilomycin A1 has no influence on fungal viability. Active pH modulation is one possible fungal strategy to change phagosome pH. In fact, *C. glabrata* is able to alkalinize its extracellular environment, when growing on amino acids as the sole carbon source *in vitro*. By screening a *C. glabrata* mutant library we identified genes important for environmental alkalinization that were further tested for their impact on phagosome pH. We found that the lack of fungal mannosyltransferases resulted in severely reduced alkalinization *in vitro* and in the delivery of *C. glabrata* to acidified phagosomes. Therefore, protein mannosylation may play a key role in alterations of phagosomal properties caused by *C. glabrata*.

## Introduction

*Candida* spp. are the most frequent causes of invasive fungal infections in the United States [1], [2], with an associated mortality rate of 30% to 50% [3]. The *Candida* species distribution has shifted in recent years: *C. albicans* remains the most frequently isolated species, but an increasing fraction of cases is caused by non-*albicans* species. Of particular concern is the emergence of *C. glabrata* as the second most frequent cause of invasive candidiasis [4]. Fungal tolerance to azole-class antifungals and the tendency of *C. glabrata* to acquire drug resistance during antifungal therapy are associated with treatment failure and death [5]–[8].

Surprisingly, and despite its medical importance, *C. glabrata* is non-lethal and elicits a low inflammatory immune response in systemic models of mouse infection, even following intravenous infection with high inocula [9]–[11]. Nevertheless, viable fungi can readily be isolated from organs of immunocompetent animals several weeks after infection, indicating that even a fully functional immune system cannot efficiently clear *C. glabrata* [11], [12]. To explain these surprising findings, we proposed that *C. glabrata* employs an immune evasion strategy, possibly via concealment in intracellular niches. Of note, we found *C. glabrata* cells associated with mononuclear cell infiltrates in all mouse organs tested (spleen, liver, brain, kidney, lung and heart), whereas no neutrophil infiltration was observed [11]. Using macrophages as a niche is a strategy that has been described for other pathogenic fungi, such as *Histoplasma capsulatum* and *Cryptococcus neoformans* [13], [14]. Our and others’ previous research showed that *C. glabrata* replicates within human and murine macrophages after phagocytosis [15]–[17], an ability which requires distinct attributes, which are only partially understood. For example, a family of glycosylphosphatidylinositol-anchored aspartyl proteases (YPS proteins) is required for survival of *C. glabrata* in macrophages [15]. Furthermore, the fungus can adapt its metabolism to starvation and can use endogenous resources to overcome nutrient limitation [15], [16]. Moreover, chromatin remodeling and DNA damage repair was shown to be crucial for viability within a macrophage phagosome [18]. Finally, by screening a set of defined *C. glabrata* mutants for reduced macrophage survival, we recently identified a series of genes required to resist intracellular killing [19]. These data support the view that immune evasion, stress resistance and nutrient acquisition are key aspects for intracellular survival.

Importantly, we found that *C. glabrata* containing phagosomes are less acidified and blocked at a late-endosomal state [17]. Mature phagolysosomes are normally strongly acidified by proton-pumping activity of vacuolar ATPase (V-ATPase). This acidic pH promotes antimicrobial effector mechanisms such as the activity of hydrolytic enzymes [20]. How phagosome maturation is blocked and acidification is prevented by *C. glabrata* and whether an immature, non-acidified phagosome is necessary for intracellular survival of the fungus is unknown.

The aim of this work was therefore to define the conditions under which non-acidified *C. glabrata* containing phagosomes are formed. We sought to find out whether this is a process, which might be dependent on *C. glabrata* initial recognition and host signaling, or whether fungal cells may actively modify their host compartment. We show that *C. glabrata* localization in non-phagolysosomal compartments is independent of the macrophage type and activation status. Further, we provide evidence for environmental alkalinization as a possible new strategy of *C. glabrata* to actively raise phagosome pH.

## Materials and Methods

### Ethics Statement

Blood was obtained from healthy human donors with written informed consent. The blood donation protocol and use of blood for this study were approved by the Jena institutional ethics committee (Ethik-Kommission des Universitätsklinikum Jena, Permission No 2207-01/08).

### Strains and Growth Conditions

Laboratory strain ATCC2001 or its GFP-expressing derivative [17] were used for characterization of macrophage – *C. glabrata* wild type interaction. *C. glabrata* mutant strains are derivatives of the laboratory strain ATCC2001, harboring auxotrophies for histidine, leucine and tryptophan (wt *hlt*Δ). Mutant strains were obtained from a novel genome-scale collection of *C. glabrata* deletion mutants. In each strain of the collection, a single open reading frame (ORF) was replaced with a bar-coded *NAT1* resistance cassette (T. Schwarzmüller, B. Cormack, K. Haynes and K. Kuchler, unpublished data). The wt *hlt*Δ strain was used for comparison. Mutant strains *anp1*Δ and *mnn11*Δ [21] were generated in a tryptophan-auxotrophic background and were thus compared to a tryptophan-auxotrophic ATCC2001 strain (wt *t*Δ).

All yeast strains used in this study were routinely grown overnight in YPD (1% yeast extract, 1% peptone, 2% glucose) at 37°C in a shaking incubator. For preparation of heat killed yeast cells, 500 µl overnight culture was incubated at 70°C for 10 min. Growth curves were monitored in a 96 well plate format by recording the absorption at 600 nm at 37°C as a function of time in an ELISA reader (Tecan Infinite 200).

### Preparation of Monocyte-Derived Macrophages (MDMs)

Human peripheral blood mononuclear cells (PBMC) were isolated by Histopaque-1077 (Sigma-Aldrich) density centrifugation from buffy coats donated by healthy volunteers. To differentiate PBMC into monocyte-derived macrophages (MDMs), 4×10^7^ PBMC were plated in RPMI 1640 media (with L-glutamine and 25 mM HEPES) (PAA Laboratories, Inc., Austria) in cell culture dishes. After 1–2 hours, non-adherent cells were washed away and 5 ng/ml M-CSF (ImmunoTools, Germany) was included in the cultures to favor the differentiation of M2-type macrophages. In selected experiments, 5 ng/ml GM-CSF was added instead to favor differentiation of M1-type macrophages. After two days at 5% CO_2_ and 37°C, medium was exchanged to RPMI 1640 containing 10% heat-treated fetal bovine serum (PAA Laboratories, Inc., Austria) and M-CSF or GM-CSF. After four more days, adherent MDMs were detached with 50 mM EDTA and plated in flat-bottom 96 and 24 well plates to give a final concentration of approximately 4×10^4^ MDMs/well and 1×10^5^ MDM/well, respectively, in RPMI 1640 with serum. Macrophage infection experiments were performed in RPMI 1640 without serum. All experiments were performed with PBMCs isolated from at least three different donors.

### Macrophage Cell Lines

The murine RAW264.7 macrophage-like cell line [22] used in this study was routinely cultured in Dulbecco’s Modified Eagle’s Medium (DMEM) with 4 mM L-glutamine and 4.5 g/l glucose (PAA Laboratories, Inc., Austria) and supplemented with 10% heat-treated fetal bovine serum (PAA Laboratories, Inc., Austria) at 37°C and 5% CO_2_. For infection experiments, RAW264.7 cells were inoculated in 6 or 24 well plates at an initial concentration of approximately 1.5×10^6^ cells/well or 2×10^5 ^cells/well, respectively, in DMEM with serum and then incubated overnight at 37°C and 5% CO_2_ to near confluency (60%–80%).

A stable J774E macrophage-like cell line expressing the subunit E (vatE) of the V1-subcomplex of V-ATPase as a green fluorescent protein fusion construct was constructed as follows. A cDNA encoding murine *vatE* was purchased from RZPD (Deutsches Ressourcenzentrum für Genomforschung GmbH, Berlin, Germany). This cDNA was PCR-amplified. The reverse primer introduced a change of the stop codon (TGA) into a serine (TCA) codon, extending the vatE coding region by six amino acid residues. The introduction of EcoRI and KpnI restriction sites by the primer pair allowed in-frame cloning of the PCR product cleaved with EcoRI and KpnI in the vector pEGFP-N1 (Addgene, Cambridge, MA). Correct in-frame cloning and point mutagenesis were confirmed by nucleotide sequencing of the product. The vatE-EGFP construct was propagated in *E. coli* and used to transfect J774E macrophages [23] by electroporation following the protocol by Schneider *et al.* [24]. Selection was done by Geneticin (0.3 mg/ml) for two weeks. Resulting clones were cultivated and frozen. Frozen stocks were thawed and transfectants cloned by dilution into 96 well plates. Five resulting clones were pooled and used for further analysis. It should be noted that the stable transfectants do not express bright vatE-EGFP in accordance with the relative scarcity of V-ATPase in the cell and regular re-selection steps are necessary. To eventually enhance the weak signal, monoclonal (not cross-reacting) murine monoclonal IgG anti-EGFP antibodies are used (see below). The resulting vatE-EGFP staining was predominantly congruent with LysoTracker staining for acidic compartments, yet not with staining of early endosome antigen-1 (EEA-1).

J774-V-ATPase-GFP cells were routinely cultured in DMEM with 4 mM L-glutamine and 4.5 g/l glucose (PAA Laboratories, Inc., Austria), supplemented with 10% heat-treated fetal bovine serum (PAA Laboratories, Inc., Austria) and 0.3 mg/ml G418 at 37°C and 5% CO_2_. For infection experiments, J774-V-ATPase-GFP cells were inoculated in 24 well plates at an initial concentration of approximately 1×10^5^ cells/well in DMEM with serum and then incubated overnight at 37°C and 5% CO_2_ to near confluency (60%–80%).

### Fluorescence Microscopy

For microscopic analysis MDM, RAW264.7 or J774-V-ATPase-GFP cells were allowed to adhere to coverslips within a 24 well plate. Overnight yeast cultures were washed with PBS, counted using a hemacytometer and adjusted to the desired concentration in cell culture medium without serum. Macrophages were then infected with yeast cells at a multiplicity of infection (MOI) of 5–10 or with latex beads or 1 µg/ml LPS. Synchronization of phagocytosis (not performed in NFκB localization study) was performed by placing the 24 well plate on ice for 30 min after infection until yeast cells settled. Unbound yeast cells were removed by washing with pre-warmed (37°C) medium, and phagocytosis was initiated by incubating at 37°C and 5% CO_2_. Co-incubation times varied between 10 and 180 min.

Acidification of the phagosomes was assessed by use of the acidotropic dye LysoTracker Red DND-99 (Life Technologies, USA). LysoTracker (diluted 1∶10,000 in cell culture medium) was added 1 h prior to infection and during co-incubation with the yeast cells. In selected experiments, 50–100 nM calcitriol (1α,25-dihydroxy vitamin D_3_, Cayman, USA) was added one night before the experiment and in parallel with LysoTracker. TROV (texas red ovalbumin, 100 µg/ml [Life Technologies, USA]) was added 2 h prior to infection, followed by a wash step and 30 min incubation in cell culture medium without TROV (chase). DQ-BSA (DQ™ Red BSA, 10 µg/ml [Life Technologies, USA]) was added 1 h prior to infection and during co-incubation with the yeast cells.

Coverslips were fixed with 4% paraformaldehyde (10 min, 37°C) at indicated time points and stained with Alexa Fluor 647-conjugated Concanavalin A (Life Technologies, USA) for 30 min. For antibody staining, macrophages were then permeabilized with 0.5% Triton-X-100 in PBS, and blocked with 2% BSA in PBS (30 min at room temperature). Cells were then incubated with a primary antibody (anti-Rab7, D95F2 XP Rabbit mAb, 1∶100, Cell Signaling, Inc., USA; anti-GFP, rat IgG2a monoclonal, 1∶500, Nacalai Tesque, Inc., Japan; NFκB (p65), 1∶100, Cell Signaling, Inc., USA) diluted in 1% BSA in PBS for 2 h, followed by incubation with a secondary Alexa Fluor-conjugated antibody (Life Technologies, USA) diluted 1∶500 in PBS. Cover slips were mounted and percentage of co-localization was calculated manually by analyzing a minimum of 100 yeast cells per well or in the case of NFκB by scoring a minimum of 100 nuclei (Leica DM5500B, Zeiss Axio Observer, Zeiss LSM).

### Western Blot Analysis

RAW264.7 macrophages were seeded in 6 well plates and infected with *C. glabrata* (viable or heat killed) at a MOI of 5 or treated with 1 µg/ml LPS. At indicated time points macrophages were lysed with 120 µl RIPA-buffer (50 mM Tris-HCl, 150 mM NaCl, 0.1% SDS, pH 7.5) containing protease and phosphatase inhibitors (complete protease inhibitor cocktail tablets, phosSTOP phosphatase inhibitor cocktail tablets [Roche, Switzerland]) for 30 min on ice. After centrifugation the supernatant was used for measurement of protein concentration (BCA protein assay kit [Thermo Fisher Scientific, Inc., USA]). 30 µg proteins were separated by SDS-PAGE and electrophoretically transferred to a nitrocellulose membrane. The membranes were blocked with 5% I-Block protein-based blocking reagent (Life Technologies, USA), solved in TBS-T (50 mM Tris-HCl [ph 7.6], 0.15 M NaCl, 0.05% Tween-20) and then incubated with primary antibodies for phosphorylated proteins in TBS-T overnight at 4°C. Antibodies specific for P-p38, P-p44/42 (P-ERK1/2), P-SAPK/JNK, P-IKKαβ, P-IκB, P-p65 were all purchased from Cell Signaling, Inc., USA. After washing three times with TBS-T, the membrane was incubated with a horseradish peroxidase-conjugated anti-rabbit or anti-mouse antibody (Santa Cruz Biotechnology, Inc., USA) followed by three washing steps. Immunoreactivity was detected by enhanced chemiluminescence (ECL Plus Western Blotting Substrate [Thermo Fisher Scientific, Inc., USA]). Membranes were then stripped with 0.5 M Tris-HCl (pH 6.7), 2% SDS and 100 mM β-mercaptoethanol for 30 min at 56°C. To ensure application of equal protein amount, membranes were again incubated with primary antibodies for unphosphorylated proteins in TBS-T overnight at 4°C (antibodies specific for p38, p44/42 (ERK1/2), SAPK/JNK, IKKα, IκB, p65 and were all purchased from Cell Signaling, Inc., USA) and detected as described above. All experiments have been performed at least in triplicate. Western Blot analysis for detection of Syk activation were performed as described elsewhere [25]. β-actin detection was included as loading control.

### Survival Assays

MDMs were seeded in 96 well plates. If necessary, cells were pre-treated with 50 µM chloroquine, 50 nM bafilomycin A1 (Sigma) or 20 µM iron nitriloacetate (FeNTA) one hour before infection. MDMs were infected at a MOI of 1 with wild type or mutant *C. glabrata* strains and incubated at 37°C and 5% CO_2_ with or without chloroquine, bafilomycin A1 or FeNTA. Survival of macrophage-internalized yeasts was assessed after 3 h or 24 h by removing non-cell-associated yeasts by washing with RPMI, subsequent lysis of MDMs with 20 µl 0.5% Triton-X-100 per well and plating lysates on YPD plates to determine colony forming units (cfus). For survival experiments comparing M1- and M2-type macrophages, macrophage-associated plus non-associated yeasts were plated and cfus were compared to wells containing yeasts only.

### Alkalinization Experiments

Liquid alkalinization-promoting medium contained 1×YNB without amino acids and ammonium sulfate (BD Bioscience, USA), 1% casamino acids (BD Bioscience, USA) and 20 mg/l phenol red. Solid alkalinization-promoting medium contained 1×YNB without amino acids and ammonium sulfate, 1% casamino acids, 2% agar and 0.01% bromocresol green. Both media were adjusted to pH 4. For experiments with triple-auxotrophic strains, histidine (0.1 g/l), leucine (0.02 g/l) and tryptophan (0.05 g/l) were added to the media.

Alkalinization in liquid media was assayed by inoculating 1×10^6^ *C. glabrata* cells/ml in a 24 well plate and incubating at 37°C while shaking at 180 rpm. pH indicator color was photographed after 20–24 hours. Growth controls were performed by measuring OD_600_ in alkalinization-promoting medium without pH indicator or in YPD medium using an ELISA reader (Tecan Infinite 200).

Alkalinization on solid media was assayed in a 96 well format with incubation at 37°C. pH indicator color was photographed after 9 hours. Growth controls were performed by monitoring colony size on solid alkalinization-promoting medium without pH indicator or on solid YPD medium.

For screening of auxotrophic *C. glabrata* deletion mutants (T. Schwarzmüller, B. Cormack, K. Haynes and K. Kuchler, unpublished data) for alkalinization defects, cells were inoculated from glycerol stocks and subcultured twice over night at 37°C in liquid YPD. Then 6 µl of the *C. glabrata* cultures were spotted on solid medium containing bromocresol green as described above. *C. glabrata* mutants that did not show a pH indicator change from green to blue, but were forming colonies on control plates, were considered as alkalinization-defective. Every assay contained a triple-auxotrophic wild type and medium alone as controls. Alkalinization defects were verified with defined inoculum cell numbers in liquid alkalinization medium containing phenol red as described above.

### Statistical Analysis

All experiments were performed at least in triplicate (n≥3). All data are reported as the mean ± SD. The data were analyzed using two-tailed, unpaired Student’s *t*-test for inter-group comparisons. For data sets based on microscopic quantification (percentage of counts), an arcsine transformation was performed prior to the t-test. A minimum of 100 yeast cells per sample or in the case of NFκB a minimum of 100 nuclei were counted. Statistical significant results were marked with a single asterisk meaning P value<0.05, double asterisks meaning P value<0.01 or triple asterisks meaning P value<0.005.

## Results

### *C. glabrata* Containing Phagosomes do not Reach the Phagolysosomal State

**Figure 1:**
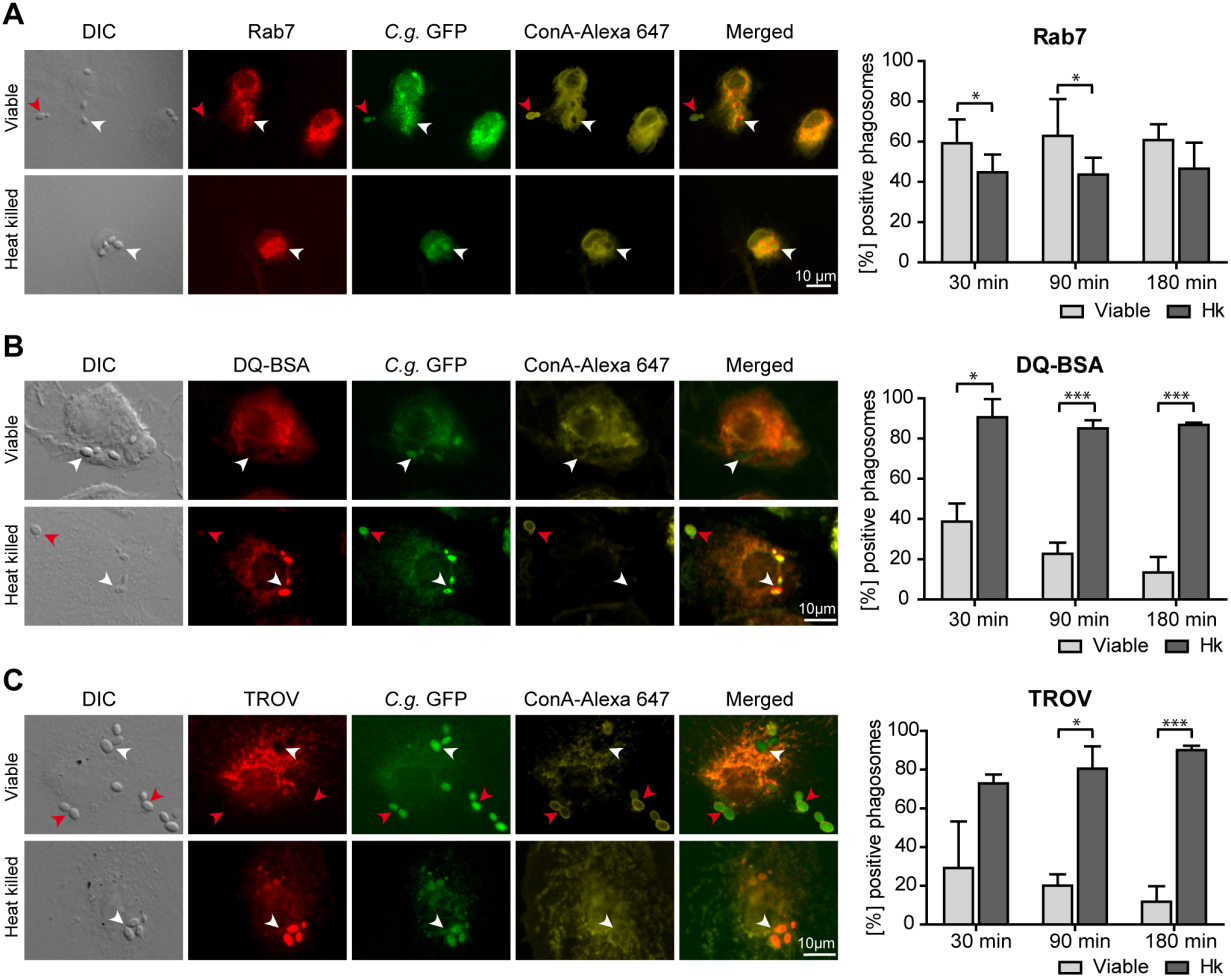
*C. glabrata* resides in non-matured macrophage phagosomes. Representative DIC and fluorescence microscopy images of viable or heat killed *C. glabrata* 90 min post infection phagocytosed by human MDMs (left panels) showing marker proteins in red, while GFP-expressing *C. glabrata* are indicated in green. To detect non-phagocytosed yeasts, samples were stained with Concanavalin A (ConA, shown in yellow). Phagocytosed yeasts are labeled with a white arrow while non-phagocytosed yeasts are marked with red arrows. Co-localization with fluorescence markers was quantified for phagosomes containing viable or heat killed (Hk) *C. glabrata* at indicated time points (right panel). (A) Phagosomes containing viable and heat killed *C. glabrata* co-localize with the late endosome marker Rab7. (B) In contrast to heat killed *C. glabrata* containing phagosomes, compartments containing viable yeasts show low phagosomal proteolytic activity as measured by co-localization with the fluorogenic protease substrate DQ-BSA. (C) Heat killed but not viable *C. glabrata* containing phagosomes acquire the lysosomal tracer texas red ovalbumin (TROV). Statistical analysis was performed comparing heat killed with viable *C. glabrata* at indicated time points (n≥3; *p<0.05, ***p<0.005 by unpaired Student’s t test).

Our previous analyses on maturation of phagosomes containing viable *C. glabrata* in macrophages revealed a compartment positive for the late endosome marker LAMP1 but less acidified than phagolysosomes containing heat killed yeasts [17]. Here we aimed at a more detailed characterization of the *C. glabrata* containing vacuole to better understand the composition of phagosomes, in which *C. glabrata* is able to survive. We therefore analyzed further markers of phagosome maturation in infected monocyte-derived macrophages (MDMs) as well as a murine macrophage-like cell line. As with LAMP1, the majority of phagosomes containing viable and heat killed yeasts acquired the small GTPase Rab7 as a marker protein of late endosomes (Fig. 1A).

DQ-BSA is a tracer for proteolytic activities. Once cleaved in acidic intracellular lysosomes, it generates a highly fluorescent product that can be monitored by microscopy. As our previous data showed viable *C. glabrata* to be localized in non-acidified phagosomes, we expected a low DQ-BSA staining for these compartments. Indeed, viable *C. glabrata* cells are located in less degradative phagosomes as compared to heat killed cells (Fig. 1B). A similar result was obtained when the fluid phase tracer texas red ovalbumin (TROV) had been chased into lysosomes. The number of TROV-positive yeast-containing phagosomes was dramatically increased for macrophages infected with heat killed as compared to viable cells (Fig. 1C). We conclude that viable *C. glabrata* containing phagosomes reach the late endosomal stage but do not fuse with lysosomes, resulting in an environment with low degradative activity. Similar data demonstrating the disparity in maturation of viable and heat killed yeast containing phagosomes were obtained with murine RAW264.7 macrophages (Fig. S1).

### Phagosome Maturation Arrest Occurs Independent of Macrophage Type, Differentiation or Activation Status and is Specific for *C. glabrata* Containing Compartments

**Figure 2:**
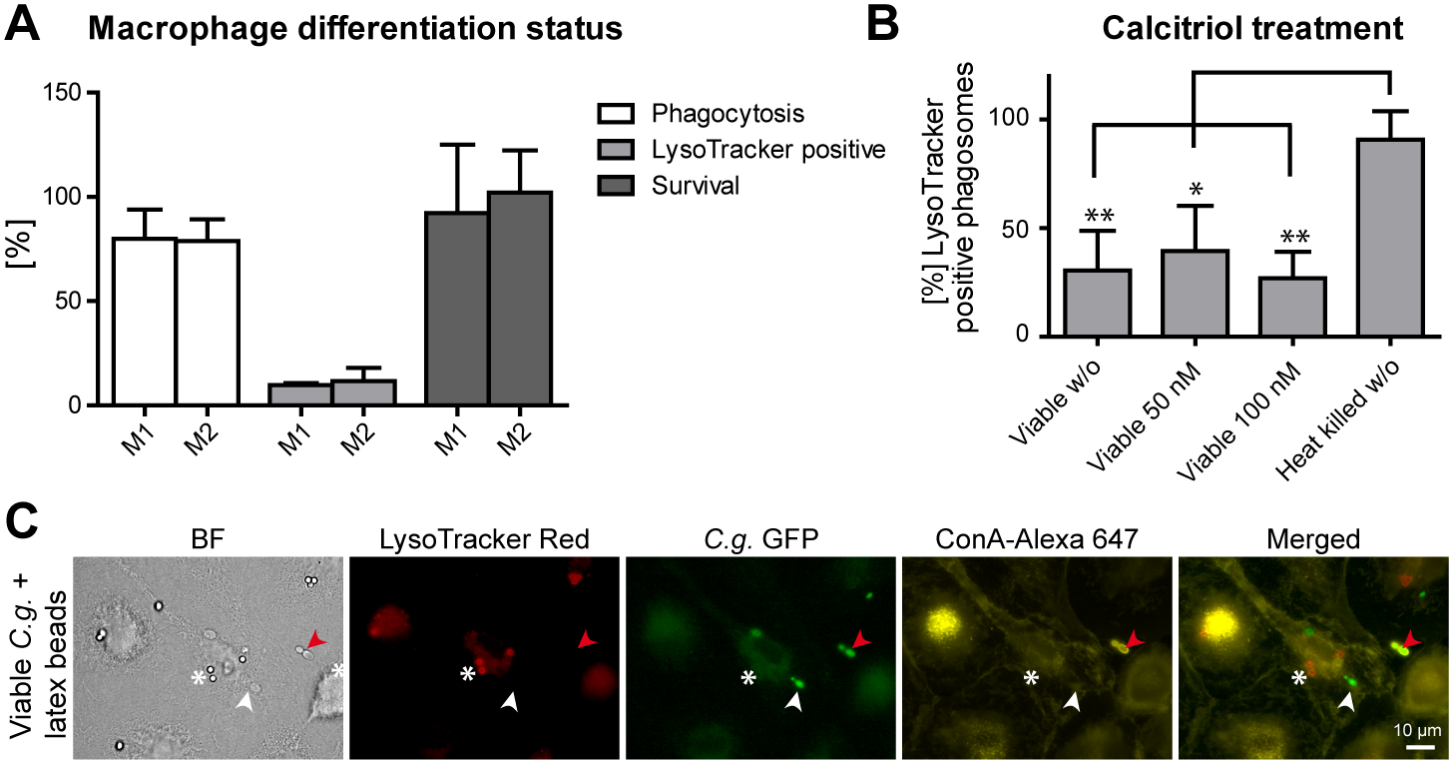
Phagosome maturation arrest occurs in different macrophage differentiation or activation states and is yeast phagosome-specific. (A) Human M1-polarized and M2-polarized MDMs do not differ in central aspects of *C. glabrata*-macrophage interaction: phagocytosis, phagosome acidification and killing. Phagocytosis (MOI of 5) was quantified microscopically by determining the percentage of internalized (Concanavalin A stain-negative) yeasts out of total yeasts after 90 min. Phagosome acidification was quantified microscopically by determining the percentage of LysoTracker-positive phagosomes after 90 min. Survival of *C. glabrata* was determined by cfu-plating of macrophage lysates after 3 h of co-incubation and comparing to yeasts incubated without macrophages. (B) Treatment with vitamin D_3_ (calcitriol) has no influence on the number of LysoTracker-positive viable *C. glabrata* containing phagosomes of human MDMs. (C) MDMs co-infected with *C. glabrata* and latex beads show a acidification defect specific to *C. glabrata* containing phagosomes (LysoTracker-negative staining; white arrow) but acidify latex bead containing phagosomes (LysoTracker-positive staining; white asterisk). Representative image 90 min post infection. GFP-expressing *C. glabrata* is indicated in green and non-phagocytosed yeasts stained with Concanavalin A (ConA) are in yellow (marked with red arrows). Statistical analysis was performed comparing M1-type with M2-type macrophages (A) or drug treated with untreated viable *C. glabrata* (B) (n≥3; *p<0.05, **p<0.01 by unpaired Student’s t test).

In the human body, macrophages change their physiology in response to environmental stimuli such as innate and adaptive immune responses. This generates different populations of macrophages with distinct functions. M1-type or classically activated macrophages are often associated with higher microbicidal activity, while M2-type or alternatively activated macrophages are more connected to regulatory functions [26]. To determine whether the initial macrophage differentiation status has an influence on *C. glabrata*-macrophage interaction, we tested whether GM-CSF stimulation, resulting in M1-polarized macrophages would enhance fungicidal activity as compared to M-CSF stimulation, resulting in M2-polarized macrophages. We detected no difference in phagocytosis rate, phagosome acidification or fungal survival in M1- or M2-polarized macrophages (Fig. 2A).

Besides cytokines, other endogenous factors can regulate macrophage functions. Vitamin D_3_ (calcitriol) is known to activate antimicrobial activity of macrophages against the intracellular pathogen *Mycobacterium tuberculosis* [27], [28]. To find out whether *C. glabrata* containing macrophages can be activated in a similar way, we tested intracellular survival of *C. glabrata* and acidification of *C. glabrata* phagosomes in calcitriol-treated macrophages. No differences between treated and untreated macrophages were observed (Fig. 2B).

Next, we sought to evaluate whether phagocytosis of *C. glabrata* by a macrophage globally modifies phagosome maturation of neighboring, non-fungal containing phagosomes in the same macrophage. We therefore analyzed phagosome acidification in macrophages that had taken up *C. glabrata* in combination with latex beads. Lack of phagosome acidification was only observed for *C. glabrata* containing phagosomes, while neighboring latex-bead containing phagosomes in the same macrophage were acidified (Fig. 2C).

In summary, *C. glabrata* persistence in macrophages in non-mature, non-acidified phagosomes is not affected by different macrophage differentiation programs and activation types, and is specific to fungus containing phagosomes.

### Viable or Heat Killed *C. glabrata* do not Evoke Different Intracellular Signaling Pathways

Recognition of ligands by receptors at the macrophage membrane activates a series of intracellular signaling pathways that lead to both, reorganization of the actin cytoskeleton, which is essential for the phagocytic uptake, and expression of immunomodulatory proteins, such as cytokines [29]. Thus, variations of intracellular signaling within macrophages may hint towards a different recognition of viable vs. heat killed *C. glabrata* cells, which may have an impact on the maturation of phagosomes. We therefore analyzed the activation of three different MAP-kinases induced upon recognition of microorganisms by macrophages [30]–[32]. Moreover, components of the nuclear factor-κB (NFκB) pathway, which have been shown to be central in the expression of immunomodulatory factors, were examined upon infection of RAW264.7 macrophages with viable or heat killed *C. glabrata* cells. A treatment with LPS served as a positive control.

**Figure 3:**
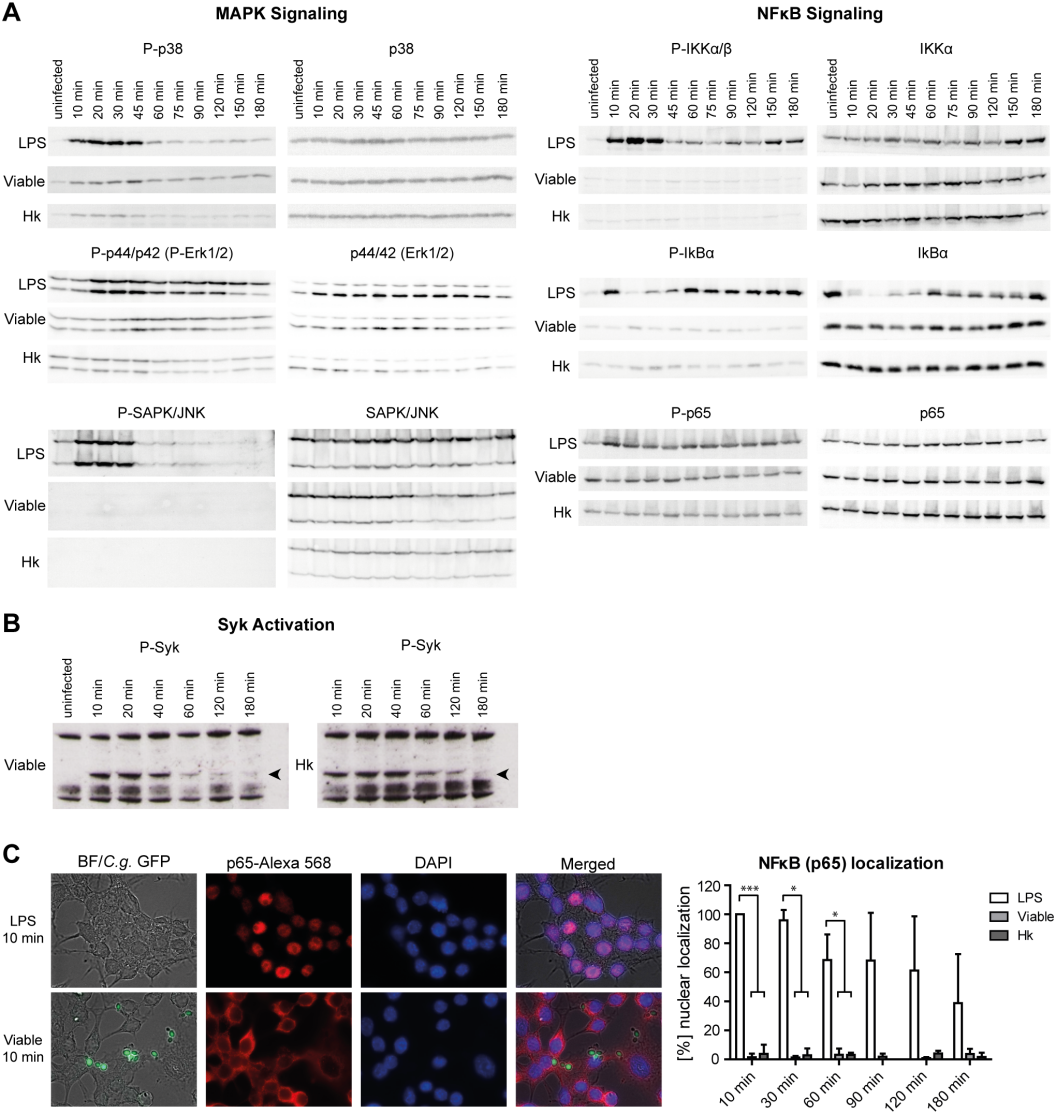
*C. glabrata* does not induce MAP-kinase or NFκB signaling cascades upon phagocytosis but activates Syk. RAW264.7 macrophages were stimulated with LPS (1 µg/ml) or infected with viable or heat killed (Hk) *C. glabrata* (MOI of 5) for indicated time points. (A) Cell lysates were subjected to Western Blot analyses by using antibodies detecting either the phosphorylated or unphosphorylated form (as a loading control) of p38, p44/42 (Erk1/2), SAPK/JNK, IKKαβ, IκBα and p65. Only LPS treatment induced changes in phosphorylation patterns of analyzed proteins. Data shown are representatives of three independent experiments. (B) Cell lysates were resolved on SDS-PAGE and membranes blotted for phosphorylated Syk (P-Syk) as described in [25] (C) Localization of the NFκB subunit p65 was analyzed by immunofluorescence microscopy. Representative pictures of macrophages treated with LPS or viable *C. glabrata* for 10 min are shown on the left site, a quantification of indicated time points on the right site. Percentage of NFκB nuclear localization was quantified for all macrophages (LPS) or for yeast-bound macrophages (viable, heat killed). While LPS induced the translocation of p65 to the nucleus, *C. glabrata* independent of its viability, did not. Statistical analysis was performed for *C. glabrata-*infected versus LPS-treated macrophages at the indicated time points (n≥3; *p<0.05, ***p<0.005 by unpaired Student’s t test).

The three major subgroups of MAP-kinases, comprising of the extracellular signal-regulated kinases (ERKs), the stress-activated protein kinases/c-jun amino-terminal kinases (SAPK/JNKs) and the p38 MAP-kinases, are all activated by phosphorylation of a common threonine-X-tyrosine regulatory motif. The activation state can be monitored by Western Blot analyses with the appropriate anti-phospho MAP-kinase antibody. As expected, LPS enhanced the phosphorylation of all three types of MAP-kinases as early as 10 to 20 min after treatment, whereas neither viable nor heat killed *C. glabrata* cells induced a strong MAP-kinase phosphorylation even at a high MOI of 5 (Fig. 3A). Only p38 was slightly activated by both cell types. Activation of the NFκB signaling involves activation of an IκB kinase (IKK) complex catalyzing a phosphorylation-induced, proteasome-mediated degradation of inhibitory protein IκB. This leads to the release and activation of the transcription factor NFκB [33]. LPS treatment induced phosphorylation of the IKKα/β catalytic subunits of the IKK complex after 10 min (Fig. 3A). As a consequence, the NFκB binding protein IκB was phosphorylated and thus degraded within 45 min of treatment, indicated by a signal reduction of the phosphorylated and unphosphorylated form. This suggests that the NFκB transcription factor is released and activated. Besides release of sequestration of NFκB in the cytoplasm, stimulus-induced phosphorylation of the p65 subunit plays a key role in activation and nuclear translocation of NFκB [33]. We therefore analyzed phosphorylation of serine 536 of the NFκB subunit p65 upon infection of macrophages with LPS or *C. glabrata*, either viable or heat killed. Compared to the uninfected control, only LPS induced a detectable increase of the phosphorylated form of p65 (Fig. 3A).

In addition to the above-mentioned pathways, signaling downstream of the β-glucan receptor dectin-1, via activation of the spleen tyrosine kinase (Syk), has recently been described to influence phagosome maturation of *C. albicans* containing vacuoles [25]. Western Blot analysis detected Syk phosphorylation immediately after both, heat killed and viable *C. glabrata* infection (Fig. 3B). Of note, activation was retained longer for heat killed cells (up to 120 min) as compared to viable cells (40–60 min). LPS treatment did not induce Syk phosphorylation (data not shown).

In addition to Western Blot analyses, immunofluorescence staining of the p65 subunit of NFκB confirmed its translocation to the nucleus of macrophages upon treatment with LPS as early as 10 min after addition (Fig. 3C). Viable or heat killed *C. glabrata*, however, did not induce a shuttling of NFκB from the cytoplasm to the nucleus at any time point investigated. Taken together, these data show that viable and heat killed yeasts do not induce a strong or differential activation of three major MAP-kinase pathways and the NFκB pathway. In contrast, Syk activation is evident and prolonged after infection with heat killed as compared to viable cells.

### Effect of Phagosome pH on *C. glabrata* Survival

**Figure 4:**
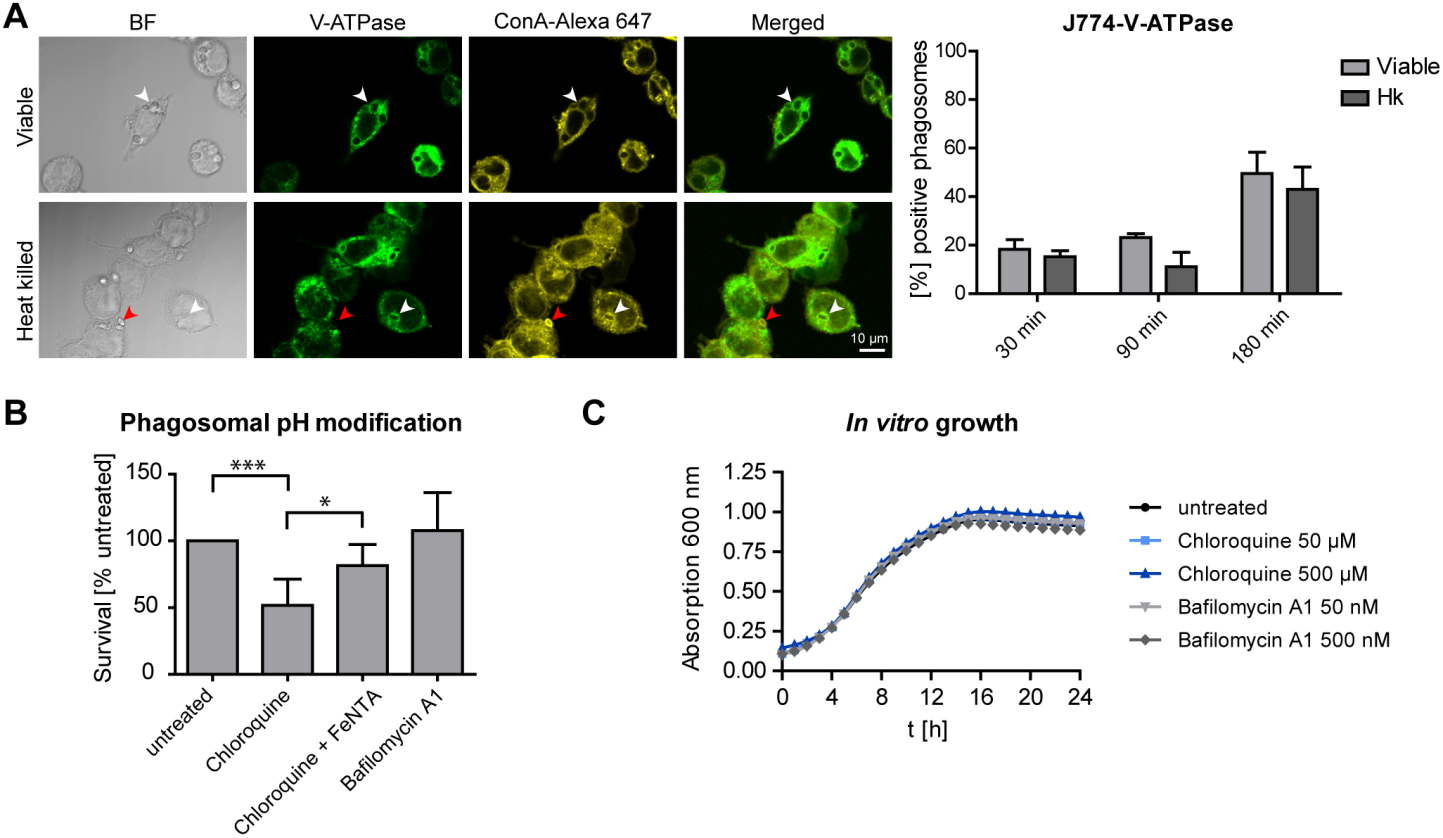
Effect of phagosome pH on *C. glabrata* survival. (A) Viable and heat killed *C. glabrata* containing phagosomes acquire similar levels of V-ATPase. Representative fluorescence microscopy images of viable or heat killed *C. glabrata* 180 min post-infection phagocytosed by murine J774E cells expressing a V-ATPase-GFP fusion protein (left panel). V-ATPase is shown in green while non-phagocytosed yeasts (stained with concanavalin A [ConA]) are indicated in yellow (marked with red arrows). Phagocytosed yeasts are labeled with white arrows. Co-localization with V-ATPase was quantified for phagosomes containing viable or heat killed *C. glabrata* at indicated time points (right panel). (B) Rising phagosome pH with chloroquine but not bafilomycin A1 reduces *C. glabrata* survival in MDMs. Survival of *C. glabrata* was determined by cfu-plating of macrophage lysates after 24 h. Co-incubation samples contained no drug (untreated), chloroquine (50 µM), chloroquine plus iron nitriloacetate (20 µM, FeNTA) or bafilomycin A1 (50 nM). (C) Chloroquine or bafilomycin A1 are not toxic to *C. glabrata in vitro*. Growth in presence of the drugs is comparable to untreated cultures. Statistical analysis was performed comparing heat killed with viable *C. glabrata* at indicated time points (A) or comparing untreated and drug-treated samples (B) (n≥3; *p<0.05, ***p<0.005 by unpaired Student’s t test).

Maturing phagosomes become increasingly acidic due to delivery of H^+^ into the phagosomal lumen via the vacuolar ATPase (V-ATPase). To elucidate whether reduced acidification of *C. glabrata* containing phagosomes may be a consequence of reduced V-ATPase accumulation on phagosome membranes, we used J774E macrophages expressing a GFP-tagged V-ATPase. Using anti-GFP antibody staining, we detected tagged V-ATPase on membranes of about 50% of viable *C. glabrata* containing phagosomes after 180 min of co-incubation, but also on acidified, heat killed yeast containing phagosomes (Fig. 4A). Thus, a reduced accumulation of V-ATPase is likely not the reason for reduced phagosome acidification.

We next sought to determine whether artificial elevation of phagosome pH or inhibition of V-ATPase activity would affect *C. glabrata* survival in macrophages. For this, we added the weak base chloroquine or the V-ATPase inhibitor bafilomycin A1 to macrophages infected with *C. glabrata*. The addition of both drugs raised the pH of heat killed yeast containing phagosomes, as observed by loss of a LysoTracker signal, but did not induce macrophage damage (data not shown) or inhibit *in vitro* growth of *C. glabrata* (Fig. 4C). Neutralizing the pH of macrophage phagosomes with chloroquine significantly reduced the survival of *C. glabrata* (Fig. 4B). However, this survival defect was rescued by the addition of FeNTA, an iron containing compound soluble at neutral pH [34] (Fig. 4B), arguing for an iron-dependent inhibitory effect of chloroquine on fungal survival.

In contrast, when adding bafilomycin A1, we observed no effect on survival of the whole population of *C. glabrata* after phagocytosis by macrophages (Fig. 4B), indicating that acidification by V-ATPase is not involved in *C. glabrata* killing. However, video microscopy of untreated RAW264.7 macrophages in presence of LysoTracker showed that a small subset of viable yeast cells was delivered to acidic phagosomes, which then resulted in degradation of the respective cells (Video S1).

Together, these findings support the view that the majority of viable *C. glabrata* cells are able to efficiently counteract V-ATPase proton pumping activity and that additional chemical inhibition of the proton pump has no impact on fungal survival.

### Environmental Alkalinization by *C. glabrata*

**Figure 5:**
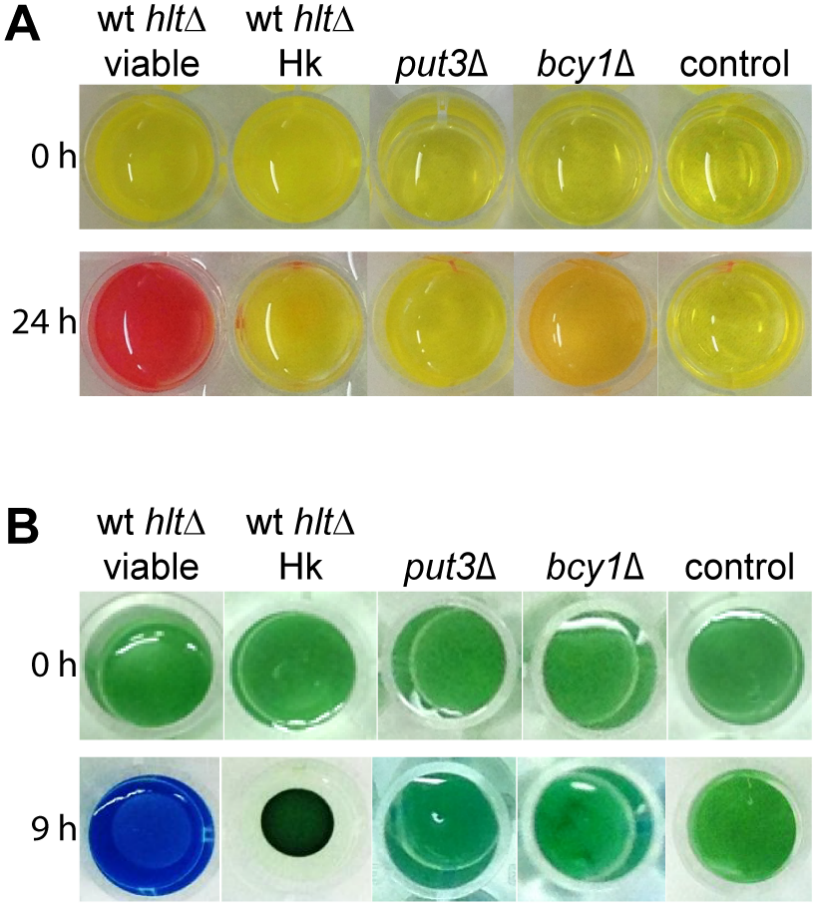
Environmental alkalinization by *C. glabrata.* (A) Viable, but not heat killed *C. glabrata* were able to alkalinize an acidic medium when grown with amino acids as the sole carbon and nitrogen source. 1×10^6^ *C. glabrata* cells/ml were inoculated in a 24 well plate with liquid YNB medium with 1% casamino acids and 20 mg/l phenol red. Within 24 h the pH rose from pH 4 to a pH above 6.8, as indicated by a color change of the pH indicator. The mutant *put3*Δ was incapable of alkalinization, the mutant *bcy1*Δ showed an intermediate phenotype of reduced alkalinization. (B) Similar results were obtained when using a solid medium (YNB with 1% casamino acids, 2% agar and 0.01% bromocresol green).

We reasoned that the lack of acidification of *C. glabrata* containing phagosomes may be due to fungal metabolic processes that actively raise the phagosome pH. We found that similar to *C. albicans*, *C. glabrata* is able to alkalinize an originally acidic minimal medium when grown with 1% casamino acids as the sole carbon and nitrogen source [35]. The pH of the medium increased from pH 4 to a pH above 6.8, as indicated by a color change of the pH indicator phenol red after 24 hours (Fig. 5A). A subsequent direct pH measurement showed a medium pH value of around 7.5. A similar result was obtained when using solid media with bromocresol green as a pH indicator (Fig. 5B). These pH changes were not observed when heat killed *C. glabrata* cells were incubated in the same media.

To identify *C. glabrata* genes involved in the *in vitro* alkalinization process, we screened a *C. glabrata* mutant library containing 647 mutant strains (T. Schwarzmüller, B. Cormack, K. Haynes and K. Kuchler, unpublished data) for alkalinization on solid alkalinization-promoting medium. Mutants that did not grow on parallel YPD plates were excluded. With this first screening round, we identified 32 deletion mutants to be deficient in environmental alkalinization. For verification, defined cell numbers of the 32 identified mutants were grown in liquid alkalinization-promoting medium and two genetically independent clones were tested. This way, the alkalinization defect was confirmed for 19 out of 32 mutants. For some of these mutants, a growth defect in YPD or alkalinization-promoting medium without pH indicator was observed (Table 1).

**Table 1:** Alkalinization-defective *C. glabrata* mutants.

Gene designation/ORF	Alkalini-zation defect^A^	Growth defectin YPD^B^	Growth defect in YNB+1% casaminoacids pH = 4^C^	LysoTrackersignal^D^	Function(based on homology to*S. cerevisiae*)
PUT3/CAGL0L09691g	**++**	**−**	**+**	**−**	transcriptional activator, regulates proline utilization genes
ADA2/CAGL0K06193g	**++**	**+**	**−**	**+**	transcription coactivator
SPT8/CAGL0F01837g	**++**	**+**	**+**	**+**	subunit of the SAGA transcriptional regulatory complex
HAP2/CAGL0H07843g	**++**	**−**	**−**	**+**	transcriptional activator, subunit of Hap2/3/4/5 CCAAT binding complex
HAP3/CAGL0J04400g	**++**	**−**	**−**	**+**	transcriptional activator, subunit of Hap2/3/4/5 CCAAT binding complex
HAP4/CAGL0K08624g	**++**	**−**	**+**	**++**	transcriptional activator, subunit of Hap2/3/4/5 CCAAT binding complex
HAP5/CAGL0K09900g	**++**	**−**	**−**	**++**	transcriptional activator, subunit of Hap2/3/4/5 CCAAT binding complex
EMI1/CAGL0K05797g	**++**	**−**	**++**	**+**	transcriptional regulator
ADR1/CAGL0E04884g	**++**	**−**	**−**	**+**	carbon source-responsive transcription factor
SIP4/CAGL0L03377g	**++**	**−**	**+**	**++**	transcriptional activator, binds carbon source-responsive elements
SNF1/CAGL0M08910g	**++**	**+**	**++**	**−**	serine/threonine protein kinase, transcription of glucose-repressed genes
BCY1/CAGL0I05236g	**+**	**++**	**+**	**−**	cAMP-dependent protein kinase
EMI5/CAGL0I08085g	**++**	**−**	**++**	**+**	succinate dehydrogenase activity
CIT1/CAGL0H03993g	**++**	**−**	**++**	**−**	citrate synthase (glyoxylate cycle)
MDH2/CAGL0E01705g	**+**	**−**	**+**	**−**	malate dehydrogenase (glyoxylate cycle, gluconeogenesis)
PCK1/CAGL0H06633g	**++**	**−**	**++**	**−**	phosphoenolpyruvate carboxykinase (gluconeogenesis)
MNN10/CAGL0K11231g	**++**	**+**	**+**	**++**	mannosyltransferase activity
KRE1/CAGL0M04169g	**+**	**+**	**−**	**++**	cell wall glycoprotein
CAGL0A01892g	**+**	**+**	**+**	**++**	uncharacterized

Listed are mutants that showed reduced *in vitro* alkalinization of phenol red containing YNB medium with 1% casamino acids as sole carbon and nitrogen source in a screen of 647 mutants. Alkalinization defects were verified in independent assays and with two independent clones.

A +reduced alkalinization (same phenotype as *bcy1*Δ in Fig. 5A), ++ strongly reduced alkalinization (same phenotype as *put3*Δ in Fig. 5A) as compared to the wild type.

B, C Growth was monitored in parallel in YPD and in YNB medium with 1% casamino acids without phenol red by measuring absorption at 600 nm. ++ strong growth defect, + weak growth defect, - unaltered growth as compared to the wild type.

D Mutants were co-incubated with MDMs for 90 min and phagosome acidification was monitored by LysoTracker staining. At least three independent microscopic fields were scored per mutant. ++ strong increase in LysoTracker signal, + medium increase in LysoTracker signal, - no change in LysoTracker signal as compared to the wild type.

### Influence of Environmental Alkalinization on Phagosome Acidification and Maturation

If environmental alkalinization is causing an active elevation of the phagosome pH, we would expect to find alkalinization-defective mutants in more acidified phagosomes as compared to the wild type. We therefore performed LysoTracker staining on macrophages incubated with the identified alkalinization-defective mutants. Indeed, out of 19 mutants, 13 strains showed a higher number of LysoTracker-positive phagosomes as compared to the wild type (Table 1).

**Figure 6:**
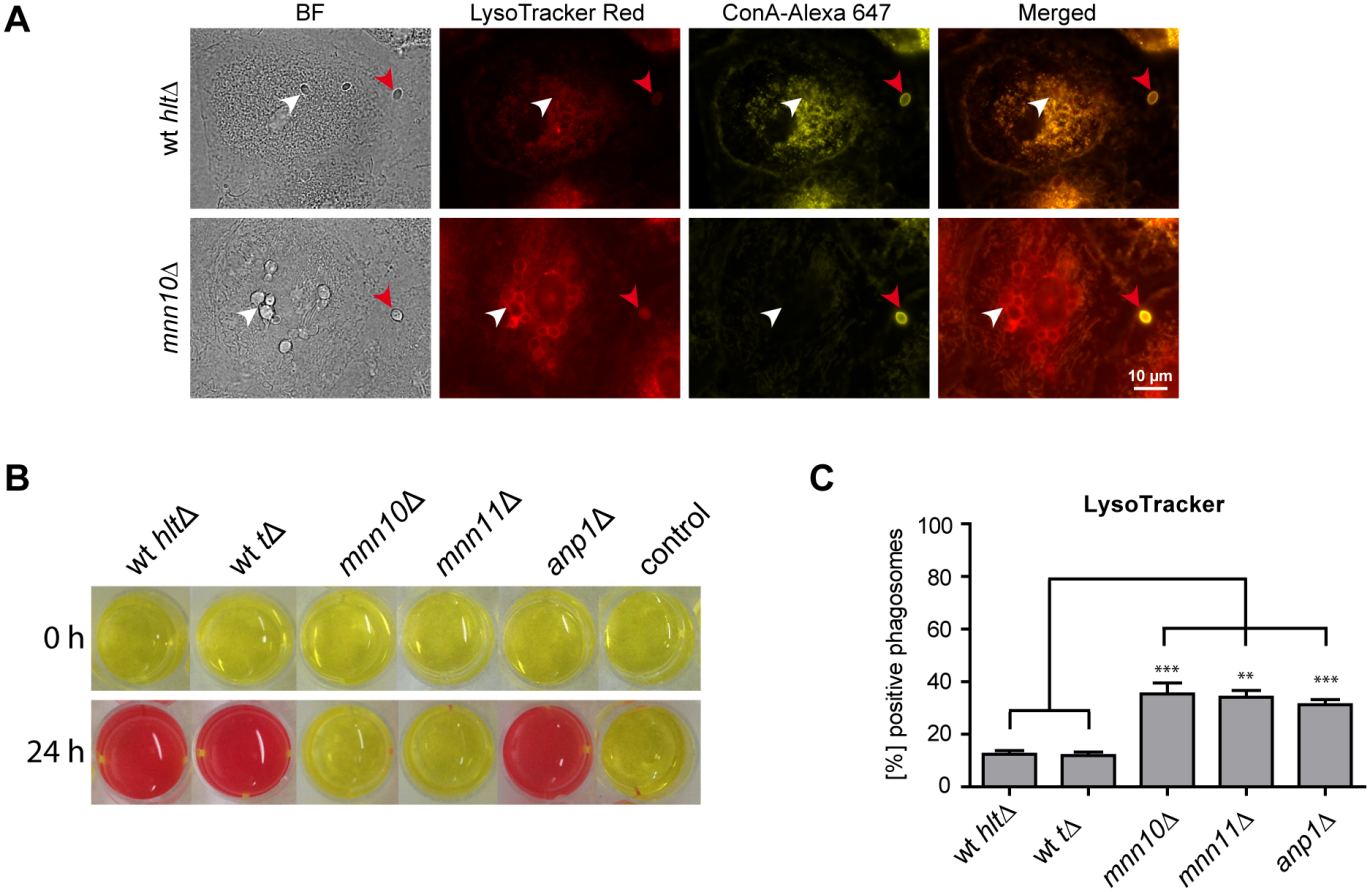
The influence of mannosyltransferases of *C. glabrata* on environmental alkalinization and acidification of phagosomes. (A) Representative fluorescence microscopy images of wild type (wt *hlt*Δ) *C. glabrata* and *mnn10*Δ mutant 90 min post infection, phagocytosed by human MDMs (left panels). LysoTracker staining is shown in red, while non-phagocytosed yeasts, stained with Concanavalin A (ConA), are shown in yellow. Phagocytosed yeasts are labeled with a white arrow while non-phagocytosed yeasts are marked with red arrows. (B) Co-localization with LysoTracker was quantified for phagosomes containing wild type (wt *hlt*Δ or wt *t*Δ) or mutant (*mnn10*Δ, *mnn11*Δ, *anp1*Δ) *C. glabrata* at 90 min post infection. Statistical analysis was performed comparing mutant with wild type *C. glabrata* (n≥3; **p<0.01, ***p<0.005 by unpaired Student’s t test). (C) *mnn10*Δ and *mnn11*Δ mutants showed severe defects in environmental alkalinization *in vitro*, while alkalinization by the *anp1*Δ mutant was comparable to isogenic wild type levels. 1×10^6^ *C. glabrata* cells/ml were inoculated in a 24 well plate with liquid YNB medium with 1% casamino acids and 20 mg/l phenol red and incubated for 24 h.

The strongest LysoTracker signal was observed for the *C. glabrata mnn10*Δ mutant (Fig. 6A). CAGL0K11231g (*MNN10*) codes for a putative α-1,6-mannosyltransferase, that is involved in glycosylation of cell wall components [36]. In contrast to wild type cells, a significantly higher percentage of *mnn10*Δ cells showed an accumulation of LysoTracker around phagocytosed yeast cells (Fig. 6B). These data suggest that *mnn10*Δ cell containing phagosomes are indeed acidified. The deletion of *MNN10* also impaired fungal survival in MDMs. Survival of the *mnn10*Δ mutant was slightly, but significantly, reduced as compared to the wild type (mutant survival 85.3% [±21.4] of wt).

Mnn10 is predicted to act in a complex with the mannosyltransferases Anp1 and Mnn11 [36]. We therefore tested *C. glabrata* mutants lacking *ANP1* and *MNN11* for *in vitro* alkalinization, phagosome acidification and survival in MDMs [21]. The *mnn11*Δ mutant phenocopied the *mnn10*Δ mutant in showing a drastic alkalinization defect (Fig. 6C) and an increased number of acidified phagosomes (Fig. 6B). In contrast, the *anp1*Δ mutant showed wild type-like alkalinization but resembled *mnn10*Δ and *mnn11*Δ phenotypes regarding phagosome acidification. Survival of *C. glabrata* in macrophages was not affected by deletion of the *MNN11* gene, while *ANP1* deletion lead to reduced survival as compared to the wild type. Survival rates, in percentage of wt, were 81.9% [±11.9] for *anp1*Δ and 111.6% [±23.3] for *mnn11*Δ.

## Discussion

Successful elimination of pathogens relies on the rapid actions of phagocytes of the innate immune system, such as macrophages, dendritic cells and neutrophils. Upon phagocytosis, the break-up of internalized microbes is carried out in phago (lyso) somes - specialized compartments in which oxidative and non-oxidative mechanisms kill and degrade microbes [37]–[39]. Therefore, immune evasion and survival strategies are critical for successful pathogens when infecting a host.

*C. glabrata* is a fungal pathogen which survives inside macrophages [15], [17], [40]. We recently showed that *C. glabrata* infection of macrophages leads to altered phagosome maturation, characterized by the arrest in a late endosomal, less acidified stage [17]. However, the mechanisms associated with the inhibited maturation and the lack of acidification were unknown. In our current study we gained further insights into the composition of *C. glabrata* containing phagosomes by analyzing markers of phagosome maturation.

Immunofluorescence microscopy demonstrated the presence of the late endosome marker Rab7, while DQ-BSA, a fluorogenic substrate for proteases, and the lysosomal tracer TROV were absent in the majority of phagosomes containing viable *C. glabrata* in MDMs and murine macrophages. These data confirmed and extended our previous results, allowing the conclusion that viable *C. glabrata* are found in phagosomes with late endosomal characteristics but with reduced acidification, reduced lysosomal fusion and low degradative activity.

Several studies have shown an impact of macrophage activation or differentiation on phagosome maturation and/or killing of intracellular pathogens. To mention a few examples, a study by Marodi *et al.* highlights the importance of INFγ to enhance clearance capacity of macrophages [41]. Further, recent studies on the fungal pathogen *Cryptococcus neoformans* or the bacterium *Chlamydia muridarum* stated an influence of macrophage differentiation: while M1 macrophages suppressed fungal and bacterial growth, M2 macrophages were less effective [42], [43]. In addition, the regulatory compound calcitriol (also called 1,25-dihydroxy vitamin D_3_), has been shown to directly promote phagocyte functions [27], [28], [44]. Pre-treatment of THP-1 macrophages with calcitriol abolished the inhibitory effect of mycobacterial cell wall glycolipid lipoarabinomannan on phagolysosome fusion [27]. Moreover, incubation of monocytes with cholecalciferol (the unhydroxylated form of vitamin D_3_) metabolites induced anti-tuberculosis activity [28].

In our previous experiments, however, we saw no influence of INFγ on replication of *C. glabrata* within MDMs, macrophage ROS production and cytokine release [17]. Differentiation of MDMs to M1 or M2 polarized macrophages did not measurably affect phagocytosis, phagosome maturation or killing of fungal cells (this study). Also, pre-treatment of MDMs with calcitriol did not enhance phagosome acidification of *C. glabrata* containing phagosomes. In addition, we found the altered fungus containing phagosome properties not only in human but also in mouse macrophages. Consequently, under the conditions investigated so far, modification of phagosome maturation seems to be a conserved feature of different types and differentiation states of *C. glabrata*-infected macrophages.

Finally, upon simultaneous infection of MDMs with viable *C. glabrata* and latex beads, phagosome acidification of vesicles containing latex beads progressed normally, while *C. glabrata* containing phagosomes within the same macrophage were not acidified. An influence of a pathogen containing vesicle on neighboring phagosomes would be expected if any secreted factor of a *C*. *glabrata* cell would affect a macrophage beyond its own compartment. For example, lipoarabinomannan of *M. tuberculosis* interferes with phagosome maturation via insertion into macrophage cell membranes [45]. Thus, our results do not support the presence of such a secreted fungal factor.

Phagocytosis is initiated by individual receptors or receptor complexes, which not only bind different ligands, but also trigger different signals. Many of these signals are controlled by kinases, including Syk and MAP-kinases that regulate phosphorylation cascades leading to effector responses including inflammatory mediators, cytokine production and antigen presentation [30], [46]–[49]. Moreover, effects of signaling mediators on maturation of phagosomes have recently been described [25]. Hence, analysis of kinase phosphorylation events in macrophages initiated by phagocytosis of *C. glabrata* could be instrumental in understanding recognition and activation of macrophages as well as alterations in phagosome maturation. Interestingly, neither viable nor heat killed yeasts induced a strong activation of three major MAP-kinases ERK1/2, SAPK/JNK or p38. In addition, even at high infectious doses, activation and translocation of NFκB, a critical transcription factor for maximal expression of many immunoregulatory molecules such as cytokines [32], [33], [50], was not observed. In line with this, previous analysis of cytokine production by MDMs revealed overall low levels of pro-inflammatory cytokines produced and no strong differences upon infection with viable or heat killed *C. glabrata* cells [17]. Thus, despite replication inside the phagosome, *C. glabrata* does not induce major signaling pathways and macrophage activation remains low. In contrast, *S. cerevisiae*, a close relative of *C. glabrata*, induces lectin- (such as dectin-1) and toll-like receptor (TLR) dependent recognition and subsequent pro-inflammatory cytokine production [51]. As determined by immunoblotting, signal transduction pathways activated in response to *S. cerevisiae* involve phosphorylation of ERK1/2, but not of p38 or SAPK/JNK [30]. Soluble β-glucan of *S. cerevisiae* leads to a significant decrease in cytosolic IκBα levels and an increase in nuclear p65 protein levels. These data and the difference to *S. cerevisiae* leads us to propose that reduced macrophage activation is a immune evasion mechanism of *C. glabrata* which contributes to persistence and low inflammatory immune responses in the systemic mouse model [11], [12].

Interestingly, we detected Syk kinase activation which was prolonged after infection with heat killed as compared to viable *C. glabrata*. When activation of Syk kinase downstream of the β-glucan receptor dectin-1 is blocked, compartments harboring *C. albicans* cells are blocked in their progression of phagosome maturation [25]. A faster release from Syk activation, by a so far unknown mechanism, may therefore be a further factor preventing full maturation of viable *C. glabrata* containing phagosomes. Syk activation further suggests dectin-1 or other Syk-coupled receptors such as dectin-2 as pattern recognition receptors mediating recognition of *C. glabrata* by macrophages. In agreement with this, a recent study has shown a role of dectin-2 for host defense against systemic *C. glabrata* infection of mice [52].

One main aim of our study was to analyze the correlation between phagosome pH, phagosome maturation and *C. glabrata* survival. Phagosomes, when undergoing maturation from early endosomal to phagolysosomal stages, accumulate the phagosomal proton pump V-ATPase, coinciding with a gradual drop in pH. This controls membrane trafficking in the endocytic pathway and may thus have an influence on phagosome maturation [53]. Consequently, the elevated pH of *C. glabrata* phagosomes may either be the cause for or the consequence of a phagosome maturation arrest.

Inhibition of phagosome acidification is a common microbial strategy to avoid destructive activities of macrophage phagosomes [54]. One possible way is the exclusion of V-ATPases from phagosome membranes to manipulate phagosome pH and maturation, as demonstrated for *M. tuberculosis* and *Rhodococcus equi* [55], [56]. This is likely not the case for *C. glabrata*, as we detected similar co-localization patterns for phagosomal V-ATPase for viable and heat killed yeast containing phagosomes. It is yet not clear whether the observed block of phagosome acidification by *C. glabrata* is a prerequisite for intracellular fungal replication or whether growth would also be possible in an acidified phagosome. In fact, *in vitro* growth of the fungus is possible at acidic pH down to pH 2 [57]. Moreover, none of the *C. glabrata* mutants identified in a large scale screening for reduced intracellular survival in MDMs lost the ability to inhibit acidification [19], which argues for pH-independent killing mechanisms. However, our observation that a small proportion of yeast cells was delivered to acidified phagosomes and degraded, suggests that an acidic phagosome at least indicates full antifungal properties.

In line with this, we showed that the proton pumping activity of V-ATPase is not required for killing of the majority of *C. glabrata* cells, as bafilomycin A1-induced inhibition of V-ATPase activity had no significant influence on overall fungal survival rates. Artificially rising phagosome pH with the weak base chloroquine, however, reduced fungal survival in macrophages. Since the reduced fungal survival rate in the presence of chloroquine was reversed by iron nitriloacetate, an iron compound soluble at neutral to basic pH, we conclude that chloroquine effects on *C. glabrata* survival are rather iron-utilization-related. A possible explanation may be that *C. glabrata* needs a slightly acidified compartment to utilize phagosomal iron sources that are crucial for intracellular survival. In presence of bafilomycin A1 that only targets V-ATPase proton pumping activity, the fungus may still be able to slightly acidify its environment to a pH value allowing iron utilization. In contrast, the weak base chloroquine may buffer such fungal activity and prevent slight acidification. A similar strategy has been suggested for intracellular survival of *H. capsulatum* [34], [58].

Besides exclusion of V-ATPase from phagosomes, there are more possible strategies to avoid phagosome acidification. First, *C. glabrata* may directly inhibit V-ATPase activity as shown for *Legionella pneumophila* [59] and other pathogens. Second, containment of viable *C. glabrata* may lead to permeabilization of phagosomal membranes, resulting in proton leakage, as observed for other fungi [60]. Third, other ion pumps that counteract V-ATPase activities, such as Na^+^-K^+^-ATPases, may be upregulated in viable yeast containing phagosomes. Finally, metabolic processes of the engulfed pathogen leading to an alkalinization of the environment, such as production of ammonia may contribute to the elevation of phagosome pH.

To test for the latter hypothesis, we set up an *in vitro* assay to determine the ability of *C. glabrata* to raise the pH of its environment. We found that environmental alkalinization by *C. glabrata* occurred within hours with similar kinetics and under similar conditions to those published by Vylkova *et al.* studying alkalinization by *C. albicans* [35]. Alkalinization took place in media lacking glucose and containing exogenous amino acids as the sole carbon source. Transcriptional profiling of *C. glabrata* phagocytosed by macrophages suggests that this yeast is exposed to similar nutritional conditions, namely glucose deprivation, inside macrophage phagosomes [15], [18]. Alkalinization by *C. albicans* relied on amino acid uptake and catabolism [35]. Mutants of *C. glabrata* lacking predicted homologous genes of the main identified *C. albicans* alkalinization factors with functions in amino acid metabolism alkalinized without any impairment (data not shown), suggesting that either other genes or other mechanisms are required for alkalinization by *C. glabrata*. In fact, *C. glabrata* shows differences in up-take and metabolism of certain amino acids as compared to *C. albicans* or *S. cerevisiae* and, for example, can grow with histidine as a sole nitrogen source by using an aromatic aminotransferase, instead of a histidinase (Brunke *et al.*, unpublished data).

A screen of a deletion mutant library (T. Schwarzmüller, B. Cormack, K. Haynes and K. Kuchler, unpublished data) for defects in alkalinization of culture medium *in vitro* identified 19 mutants. Of these, 13 mutants co-localized more frequently with LysoTracker in MDMs as compared to the wild type, indicating a possible correlation between the potential for environmental alkalinization and the elevation of phagosome pH. For most of these mutants a more or less pronounced growth defect in complete and/or minimal medium was observed, suggesting a physiological activity to be necessary to grow and alkalinize under the conditions used.

Interestingly, single mutants lacking all four components of the *HAP* complex, a heteromeric transcriptional regulator with a complex position in the global transcriptional regulation of the cell, showed up in the screening (Table 1). The HAP complex was originally identified as regulator of the ‘diauxic shift’ of *S. cerevisiae*, a reprogramming of respiratory metabolism when yeasts adapt to glucose-limiting conditions [61]. Additionally, mutants lacking genes encoding the protein kinase Snf1 and its target, the transcriptional activator Sip4, were identified. Both proteins play a role in expression of glucose-repressed genes in response to glucose deprivation [62]. Moreover, the lack of glucose is reflected by the appearance of mutants, which lack genes involved in the glyoxylate cycle and gluconeogenesis (*cit1*Δ, *mdh2*Δ; *pck1*Δ). Thus, metabolic processes that enable *C. glabrata* to adapt to nutrient limitation are vital to grow in the alkalinization medium, which consequentially raises the extracellular pH. Also, the functional divergence of alkalinization-defective mutants identified suggests that more than one distinct pathway may be involved raising extracellular pH in *C. glabrata*.

Thirteen out of 19 alkalinization-defective mutants were more frequently found in LysoTracker-positive phagosomes, suggesting that environmental alkalinization enables *C. glabrata* to actively modify phagosome pH after macrophage phagocytosis. Similarly, *C. albicans* has recently been shown to neutralize the macrophage phagosome [63]. The *C. glabrata* mutant with the strongest LysoTracker phenotype identified in our study was *mnn10*Δ, lacking a putative Golgi-localized α-1,6-mannosyltransferase. As in *S. cerevisiae*, Mnn10 is believed to act in an α-1,6-mannosyltransferase complex with Anp1 and Mnn11 on the extension of N-linked mannose backbones in *C. glabrata* [21], [36]. In our study, alkalinization and phagosome acidification phenotypes of the *mnn10*Δ and *mnn11*Δ mutants were similar, hinting towards a functional connection and possibly a redundancy of Mnn10 and Mnn11 in *C. glabrata*. Thus, Mnn10 and Mnn11-related α-1,6-mannosyltransferase functions in environmental alkalinization may enable *C. glabrata* to elevate the phagosome pH in macrophages. In this context, Mnn10 and Mnn11 glycosylation activities may be important for secretion and/or functionality of either general fungal proteins that ensure fitness and physiological activity of *C. glabrata*, of alkalinization-specific proteins or of other proteins that counteract a drop in phagosome pH. In *S. cerevisiae*, *MNN10* and *MNN11* deletion has been shown to cause a hyper-secretory phenotype [64]. Another possibility, however, would be an alkalinization-independent effect by Mnn10- and Mnn11-mediated surface modifications that influence initial recognition of *C. glabrata* by macrophages. Such an effect on phagosome pH may be also an explanation for the observed *anp1*Δ phenotype. *ANP1* seems to be dispensable for environmental alkalinization *in vitro*, while still having an influence on phagosome acidification. In addition, our data suggest an alkalinization-independent function of Anp1 in macrophage survival.

Finally, the fact that *MNN10* deletion reduced the ability of *C. glabrata* to survive in macrophages suggests that Mnn10 functions in alkalinization and phagosome modification affect the intracellular fate of *C. glabrata* in macrophages. The wild type-like survival of a *mnn11*Δ mutant may argue for a redundancy of functions among the different α-1,6-mannosyltransferases in *C. glabrata*. In addition, further fungal attributes have been shown to affect intracellular survival of *C. glabrata* in macrophages [15], [16], [19].

In conclusion, we have identified protein mannosylation as an important fungal attribute for *C. glabrata* to modify phagosome acidification. Moreover, to our knowledge this is the first study to show rapid environmental alkalinization by *C. glabrata.* This activity may at least be one strategy of *C. glabrata* to raise phagosome pH in order to create a less hostile compartment for intracellular replication.

## Supporting Information

Figure S1 ***C. glabrata*** **resides in non-matured macrophage phagosomes of murine macrophages.** Murine RAW264.7 macrophages were infected with viable or heat killed (Hk) *C. glabrata*, followed by fluorescence staining for the lysosomal tracer texas red ovalbumin (TROV), the fluorogenic protease substrate DQ-BSA or the acidotropic dye LysoTracker. Co-localization with fluorescence markers was quantified for yeast containing phagosomes at indicated time points. Heat killed but not viable *C. glabrata* containing phagosomes acquire the lysosomal tracer TROV, show high phagosomal proteolytic activity as measured by co-localization with DQ-BSA, and co-localize with LysoTracker. Statistical analysis was performed comparing heat killed with viable *C. glabrata* at indicated time points (n = 3; *p<0.05, **p<0.01, ***p<0.005 by unpaired Student’s t test).
(TIF)

Video S1 ***C. glabrata*** **replicates in LysoTracker negative compartments.** RAW264.7 macrophages were stained with LysoTracker Red (diluted 1∶10.000 in cell culture medium) 1 h prior to infection and during co-incubation with viable *C. glabrata* cells. After infection (MOI of 4) time-lapse microscopy was started and pictures were taken every 5 min at a magnification of 630×. While the majority of yeasts resides in LysoTracker negative compartments and replicates, killing of yeast cells and subsequent LysoTracker acquisition can be observed within a few macrophages (red arrows 1–3). In macrophage 2, yeast replication in close proximity to a killed *C. glabrata* cell is shown.
(AVI)
