# Supplementary figures and images for: Identification of Candida glabrata Genes Involved in pH Modulation and Modification of the Phagosomal Environment in Macrophages
Source: PLoS One. 2014 May 1;9(5):e96015. doi: 10.1371/journal.pone.0096015 (PMC4006850; doi:10.1371/journal.pone.0096015)

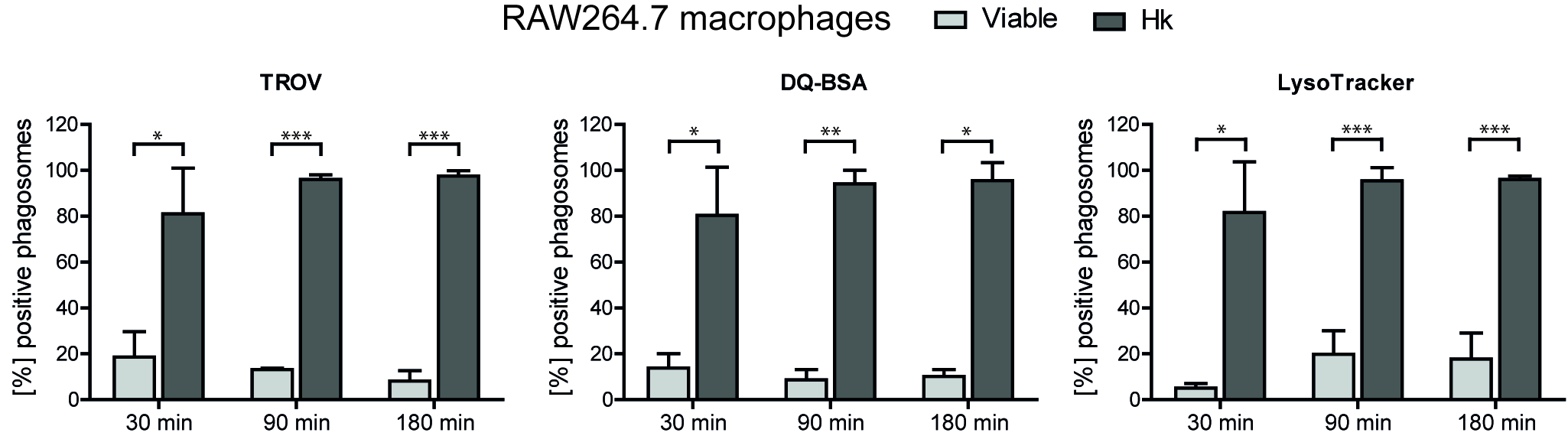

Supplement: Figure S1 — C. glabrata resides in non-matured macrophage phagosomes of murine macrophages. Murine RAW264.7 macrophages were infected with viable or heat killed (Hk) C. glabrata, followed by fluorescence staining for the lysosomal tracer texas red ovalbumin (TROV), the fluorogenic protease substrate DQ-BSA or the acidotropic dye LysoTracker. Co-localization with fluorescence markers was quantified for yeast containing phagosomes at indicated time points. Heat killed but not viable C. glabrata containing phagosomes acquire the lysosomal tracer TROV, show high phagosomal proteolytic activity as measured by co-localization with DQ-BSA, and co-localize with LysoTracker. Statistical analysis was performed comparing heat killed with viable C. glabrata at indicated time points (n = 3; *p<0.05, **p<0.01, ***p<0.005 by unpaired Student’s t test). (TIF) [file pone.0096015.s001.tif]
